# Supplementary material for: The Discovery of Selective Protein Arginine Methyltransferase 5 Inhibitors in the Management of β-Thalassemia through Computational Methods
Source: Molecules. 2024 Jun 4;29(11):2662. doi: 10.3390/molecules29112662 (PMC11173459; doi:10.3390/molecules29112662)
Supplement: Supplementary file 1 [file molecules-29-02662-s001.zip › molecules-2971566-supplementary.pdf]

Supplementary Information

Table S1. The table presents the results of various interactions of TOP1 and TOP2 with PRMT5 attained during docking studies.

Ligand	Hydrogen-forming and hydrophobic interaction	Distance Å	Category	Types
**TOP1**	B:UNL1:H - A:SAM701:O	2.71061	Hydrogen Bond	Conventional Hydrogen Bond
	B:UNL1:H - A:TYR334:OH	2.95993	Hydrogen Bond	Conventional Hydrogen Bond
	B:UNL1:H - A:SAM701:OXT	2.85945	Hydrogen Bond	Conventional Hydrogen Bond
	B:UNL1:H - A:GLU444:OE2	2.66613	Hydrogen Bond	Conventional Hydrogen Bond
	B:UNL1:H - A:LEU437:O	2.57454	Hydrogen Bond	Conventional Hydrogen Bond
	A:GLY438:CA - B:UNL1:O	3.73667	Hydrogen Bond	Carbon Hydrogen Bond
	B:UNL1:C - A:SER578:O	3.77041	Hydrogen Bond	Carbon Hydrogen Bond
	A:SAM701:CE - B:UNL1	3.92763	Hydrophobic	Pi-Sigma
	A:SAM701:CE - B:UNL1	3.47106	Hydrophobic	Pi-Sigma
	A:SAM701:SD - B:UNL1	4.22414	Other	Pi-Sulfur
	B:UNL1:S - A:PHE327	5.90361	Other	Pi-Sulfur
	A:PHE327 - B:UNL1	5.46008	Hydrophobic	Pi-Pi Stacked
	B:UNL1 - A:PHE327	3.91511	Hydrophobic	Pi-Pi Stacked
	A:VAL503 - B:UNL1	4.70012	Hydrophobic	Alkyl
	A:TYR304 - B:UNL1	5.13284	Hydrophobic	Pi-Alkyl
	A:TYR304 - B:UNL1:C	4.16201	Hydrophobic	Pi-Alkyl
	A:PHE327 - B:UNL1	4.81064	Hydrophobic	Pi-Alkyl
	A:PHE580 - B:UNL1	4.49048	Hydrophobic	Pi-Alkyl
	A:PHE580 - B:UNL1:C	4.97041	Hydrophobic	Pi-Alkyl
	B:UNL1 - A:LEU319	5.46008	Hydrophobic	Pi-Alkyl
**TOP2**	B:UNL2:H - A:TYR334:OH	2.87195	Hydrogen Bond	Conventional Hydrogen Bond
	B:UNL2:H - A:TYR334:OH	2.63923	Hydrogen Bond	Conventional Hydrogen Bond
	B:UNL2:H - A:SAM701:OXT	2.1576	Hydrogen Bond	Conventional Hydrogen Bond
	B:UNL2:H - A:LEU437:O	2.39261	Hydrogen Bond	Conventional Hydrogen Bond

B:UNL2:H - A:PHE577:O	2.41793	Hydrogen Bond	Conventional Hydrogen Bond
A:GLU435:OE2 - B:UNL2	4.94875	Electrostatic	Pi-Anion
A:SAM701:CE - B:UNL2	3.74089	Hydrophobic	Pi-Sigma
A:SAM701:CE - B:UNL2	3.46064	Hydrophobic	Pi-Sigma
A:SAM701:SD - B:UNL2	4.03251	Other	Pi-Sulfur
B:UNL2:S - A:PHE327	5.11293	Other	Pi-Sulfur
A:PHE327 - B:UNL2	4.45421	Hydrophobic	Pi-Pi Stacked
A:PHE580 - B:UNL2	4.7704	Hydrophobic	Pi-Pi Stacked
B:UNL2 - A:PHE327	3.98128	Hydrophobic	Pi-Pi Stacked
B:UNL2 - A:PHE580	4.34229	Hydrophobic	Pi-Pi Stacked
A:TYR304 - B:UNL2	4.78216	Hydrophobic	Pi-Pi T-shaped
A:PHE327 - B:UNL2	4.75143	Hydrophobic	Pi-Pi T-shaped
B:UNL2 - A:PHE327	4.91612	Hydrophobic	Pi-Pi T-shaped

UNL1- TOP1, UNL2-TOP2

Table S2. The selected drug compounds ' off-target effects (biological effects) were obtained using the PASS server.

Activity	3XV		TOP1		TOP2	
	Pa	Pi	Pa	Pi	Pa	Pi
Antiviral (HIV)	0.180	0.040	0.193	0.033	0.260	0.014
Antiviral (Hepatitis B)	0.476	0.006	0.379	0.018	0.477	0.006
Antiviral (Herpes)	0.513	0.008	0.540	0.006	0.539	0.006
Antineoplastic antimetabolite	0.765	0.003	0.495	0.011	0.678	0.005
Anticarcinogenic	0.417	0.028	0.243	0.086	0.316	0.052
Antimetastatic	0.414	0.042	0.488	0.022	0.337	0.067
Antineoplastic	0.485	0.076	0.305	0.149	0.337	0.132
Antineoplastic (non-Hodgkin's lymphoma)	0.550	0.023	0.507	0.034	0.568	0.019
Antineoplastic (solid tumors)	0.393	0.040	0.284	0.104	0.309	0.083
Antineoplastic (lymphoma)	0.287	0.026	0.198	0.056	0.233	0.039
Antineoplastic (multiple myeloma)	0.298	0.080	0.252	0.149	0.255	0.144
Analgesic stimulant	0.371	0.009	0.267	0.074	0.342	0.016
Antiischemic	0.346	0.034	0.359	0.030	0.227	0.112
Antiarrhythmic	0.333	0.023	0.265	0.038	0.139	0.119
Metabolic disease treatment	0.395	0.013	0.260	0.040	0.261	0.040
Proliferative diseases treatment	0.549	0.014	0.375	0.031	0.523	0.017
Vasculitis treatment	0.396	0.005	0.311	0.009	0.367	0.006
Respiratory distress syndrome treatment	0.367	0.013	0.013	0.032	0.373	0.012
Gout treatment	0.350	0.005	0.274	0.018	0.325	0.007
Myocardial ischemia treatment	0.169	0.042	0.221	0.024	0.109	0.091
Liver cirrhosis treatment	0.143	0.011	0.138	0.014	0.140	0.013
Biliary tract disorders treatment	0.270	0.068	0.221	0.119	0.233	0.104

Pa - probability "to be active", Pb - probability "to be inactive".

Table S3. The table presents the results of various interactions of TOP1, TOP2 and 3XV with PRMT5 attained at different time interval.

Name	Time (ns)	Interaction	Distance (Å)	Category
**PRMT5-3XV**	100	A:LEU312:N - C:3XV639:O3'	2.59124	Conventional Hydrogen Bond
		C:3XV639:O3' - A:GLU444:OE1	2.53273	Conventional Hydrogen Bond
		C:3XV639:HO2' - A:GLU444:OE2	1.46854	Conventional Hydrogen Bond
		C:3XV639:C2 - A:ASP317:O	3.71786	Carbon Hydrogen Bond
		A:LEU319:CD2 - C:3XV639	3.7571	Pi-Sigma
		C:3XV639 - A:PRO314	4.2896	Pi-Alkyl
		C:3XV639 - A:LEU319	4.48639	Pi-Alkyl
		C:3XV639 - A:PRO314	4.68343	Pi-Alkyl
	200	A:LEU312:N - C:3XV639:O3'	2.77442	Conventional Hydrogen Bond
		A:SER439:N - C:3XV639:O2'	2.79072	Conventional Hydrogen Bond
		C:3XV639:O3' - A:GLU444:OE2	2.68572	Conventional Hydrogen Bond
		C:3XV639:HO2' - A:GLU444:OE2	1.51056	Conventional Hydrogen Bond
		C:3XV639:H61 - B:SAM638:O4'	2.36442	Conventional Hydrogen Bond
		C:3XV639:H62 - A:GLU435:O	2.26918	Conventional Hydrogen Bond
		C:3XV639:C2 - A:ASP317:O	3.69743	Carbon Hydrogen Bond
		C:3XV639 - A:PRO314	4.44265	Pi-Alkyl
		C:3XV639 - A:LEU319	4.97158	Pi-Alkyl
		C:3XV639 - A:PRO314	4.27301	Pi-Alkyl
		C:3XV639 - A:LEU319	5.41761	Pi-Alkyl
**PRMT5-TOP1**	**100**	A:LEU312:HN - C:TOP1639:N4	2.85079	Conventional Hydrogen Bond
		A:PHE580:HN - C:TOP1639:O2	1.97732	Conventional Hydrogen Bond
		C:TOP1639:H26 - A:PHE580:O	2.44201	Conventional Hydrogen Bond
		C:TOP1639:H27 - A:GLU444:OE1	2.07779	Conventional Hydrogen Bond
		C:TOP1639:H22 - A:SER578:O	2.50481	Carbon Hydrogen Bond
		C:TOP1639:H23 - A:SER310:O	2.54757	Carbon Hydrogen Bond
		A:THR323:CG2 - C:TOP1639	3.87045	Pi-Sigma
		A:VAL503:CG2 - C:TOP1639	3.8163	Pi-Sigma
		A:PHE580 - C:TOP1639	4.8046	Pi-Pi Stacked
		C:TOP1639 - A:PHE580	3.99278	Pi-Pi Stacked
		A:PRO314 - C:TOP1639	5.37264	Alkyl
		C:TOP1639 - A:LEU312	5.1748	Alkyl
		C:TOP1639 - A:LEU312	5.10447	Pi-Alkyl
		C:TOP1639 - A:VAL326	5.40527	Pi-Alkyl
	**200**	C:TOP1639:H24 - A:GLN322:OE1	2.91014	Conventional Hydrogen Bond
		C:TOP1639:H26 - A:GLY438:O	1.94854	Conventional Hydrogen Bond
		C:TOP1639:H27 - A:GLY438:O	2.15678	Conventional Hydrogen Bond
		A:TRP579:CA - C:TOP1639:O2	3.54872	Carbon Hydrogen Bond
		C:TOP1639:H23 - A:SER310:O	2.20057	Carbon Hydrogen Bond
		C:TOP1639:H21 - A:SER578:O	2.46349	Carbon Hydrogen Bond
		C:TOP1639:H19 - C:TOP1639:N4	2.80877	Carbon Hydrogen Bond

		A:THR323:CG2 - C:TOP1639	3.64719	Pi-Sigma
		C:TOP1639:S1 - A:PHE327	4.96925	Pi-Sulfur
		C:TOP1639 - A:PHE580	4.34929	Pi-Pi T-shaped
		A:LEU312 - C:TOP1639	5.05523	Alkyl
		C:TOP1639 - A:VAL326	5.0178	Pi-Alkyl
**PRMT5-TOP2**	**100**	C:TOP2639:H16 - A:SER578:O	1.67454	Conventional Hydrogen Bond
		C:TOP2639:H15 - A:GLU444:OE2	1.65593	Conventional Hydrogen Bond
		A:SER439:CB - C:TOP2639:O3	3.63307	Carbon Hydrogen Bond
		C:TOP2639:H1 - A:TYR304:O	2.58271	Carbon Hydrogen Bond
		C:TOP2639:H7 - A:SER578:O	3.0148	Carbon Hydrogen Bond
		C:TOP2639:H8 - A:GLU444:OE2	2.54851	Carbon Hydrogen Bond
		C:TOP2639:H9 - A:SER578:O	2.50188	Carbon Hydrogen Bond
		C:TOP2639:H10 - A:GLU444:OE1	2.18314	Carbon Hydrogen Bond
		B:SAM638:SD - C:TOP2639	4.56841	Pi-Cation
		A:GLN309:HE21 - C:TOP2639	2.04051	Pi-Donor Hydrogen Bond
		B:SAM638:SD - C:TOP2639	4.16046	Pi-Sulfur
		A:PHE580 - C:TOP2639	5.05573	Pi-Pi Stacked
		C:TOP2639 - A:PHE580	5.39824	Pi-Pi Stacked
		C:TOP2639 - A:PHE327	5.81691	Pi-Pi T-shaped
		A:VAL503 - C:TOP2639	4.66662	Alkyl
		C:TOP2639 - A:LEU312	5.30765	Alkyl
		A:PHE580 - C:TOP2639	4.54948	Pi-Alkyl
		C:TOP2639 - A:LEU319	5.06417	Pi-Alkyl
	**200**	PHE580:HN - TOP2:O3	2.68747	Conventional Hydrogen Bond
		C:TOP2639:H15 - A:GLU444:OE2	1.60153	Conventional Hydrogen Bond
		C:TOP2639:H8 - A:GLU444:OE1	2.48685	Carbon Hydrogen Bond
		C:TOP2639:H10 - A:GLU444:OE2	2.52218	Carbon Hydrogen Bond
		A:GLN309:HE21 - C:TOP2639	2.77132	Pi-Donor Hydrogen Bond
		A:GLN309:HE21 - C:TOP2639	2.56492	Pi-Donor Hydrogen Bond
		C:TOP2639:H13 - A:TYR324	3.06439	Pi-Donor Hydrogen Bond
		B:SAM638:SD - C:TOP2639	4.40889	Pi-Sulfur
		A:PHE327 - C:TOP2639	5.63262	Pi-Pi T-shaped
		A:VAL503 - C:TOP2639	4.22903	Alkyl
		A:PHE580 - C:TOP2639	4.9011	Pi-Alkyl
		C:TOP2639 - A:LEU312	5.14319	Pi-Alkyl

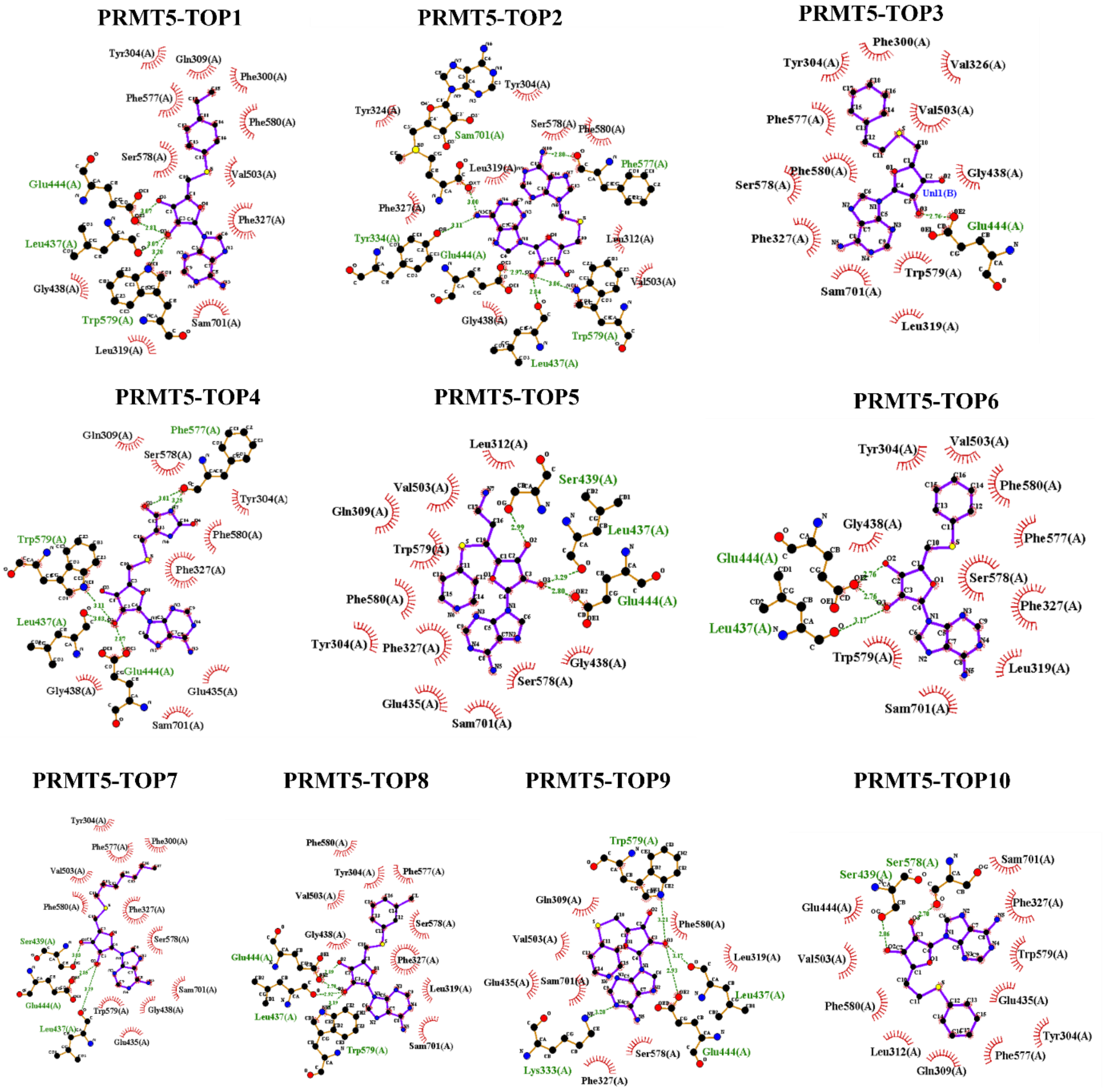

Figure S1. 2D Ligplot showing the molecular interactions of TOP10 compounds docked in the PRMT5 active site.

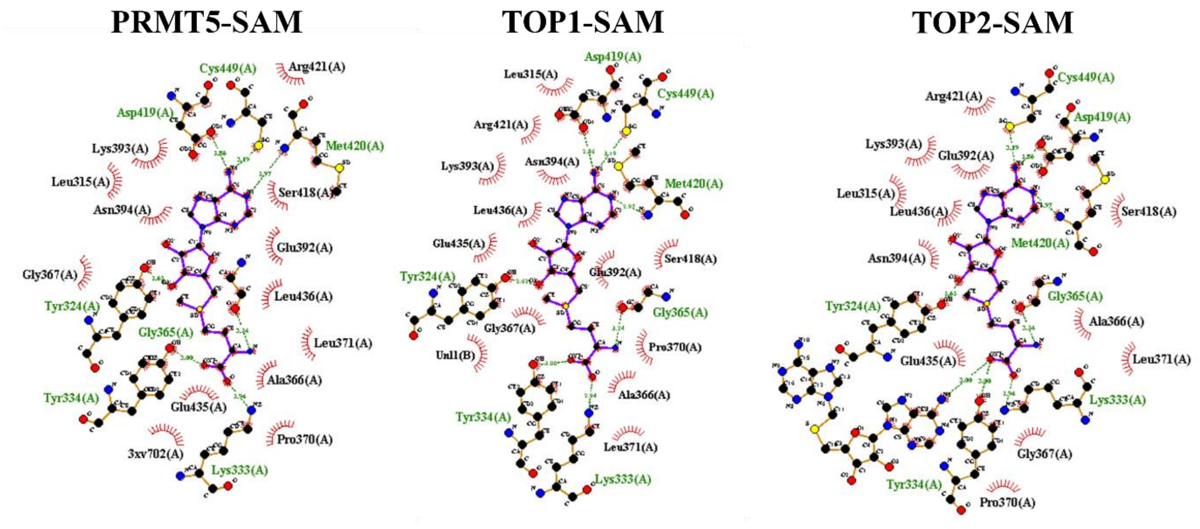

Figure S2. 2D Ligplot showing the molecular interactions of SAM in the PRMT5 active site

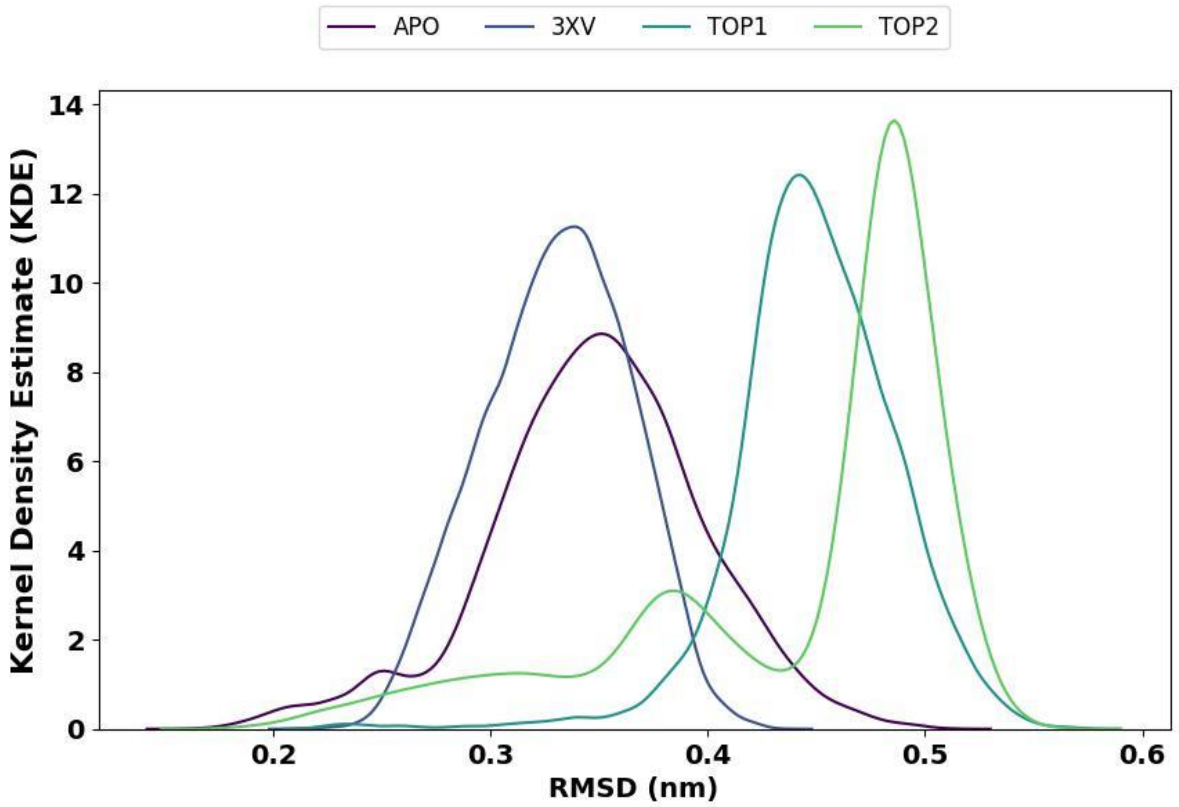

Figure S3. Kernel Density Estimate for the probability distribution of all systems. The violet color indicates the APO form, the blue color indicates the 3XV, and the Dark green color indicates the TOP1. The light green color indicates the TOP2.

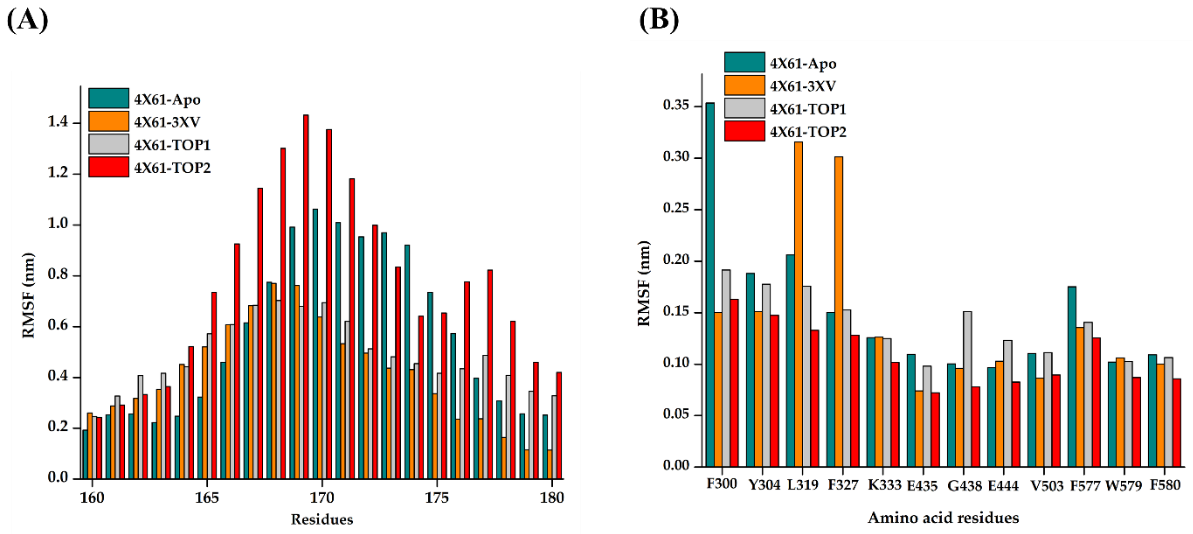

Figure S4. (A) Bar graph showing the RMSF values of the flexible regions observed during simulation. **(B)** Bar graph showing the RMSF values of the active site residues having interaction with ligands.

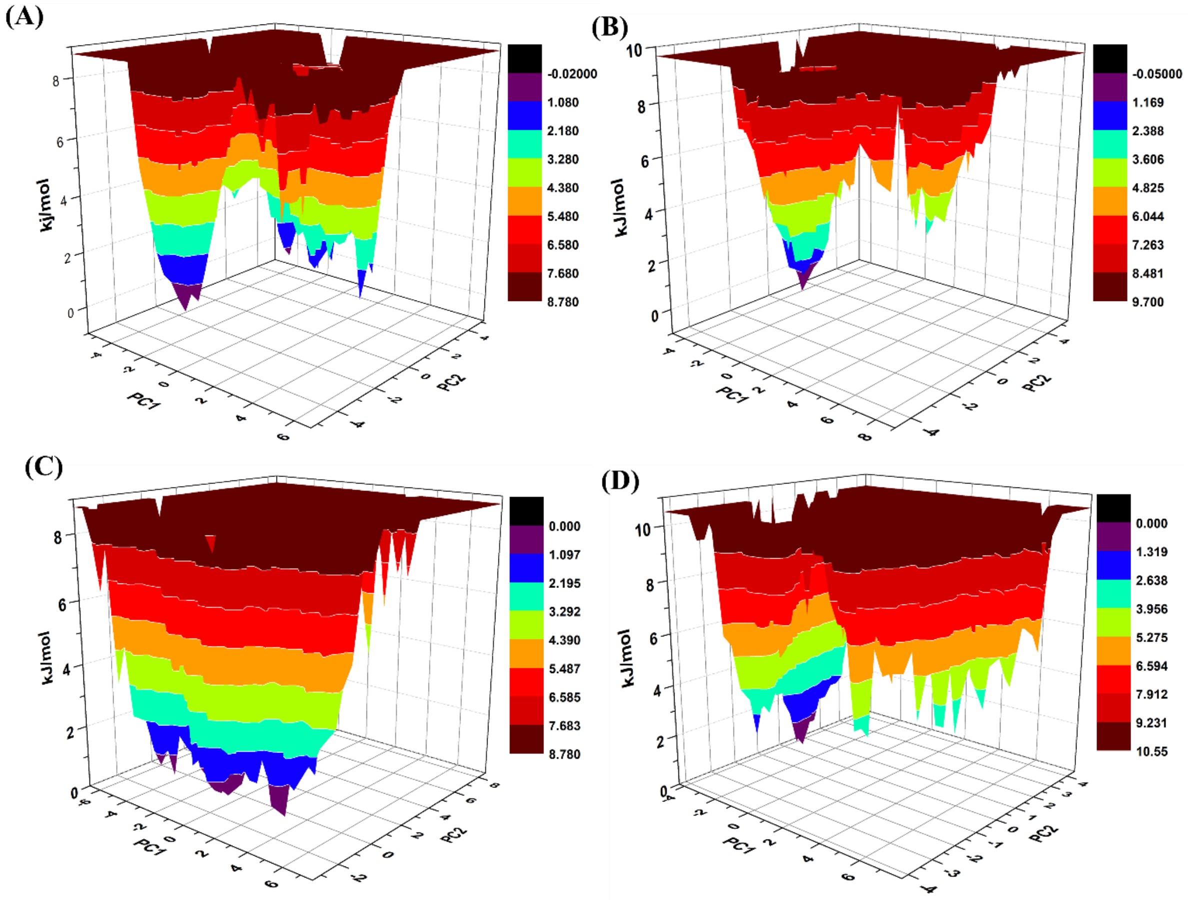

Figure S5. (A) 3D- contour map showing the FEL of apo-form **(B)** 3D- contour map showing the FEL of 3XV-PRMT5 complex. **(C)** 3D- contour map showing the FEL of TOP1-PRMT5 complex. **(D)** 3D- contour map showing the FEL of TOP2-PRMT5 complex.

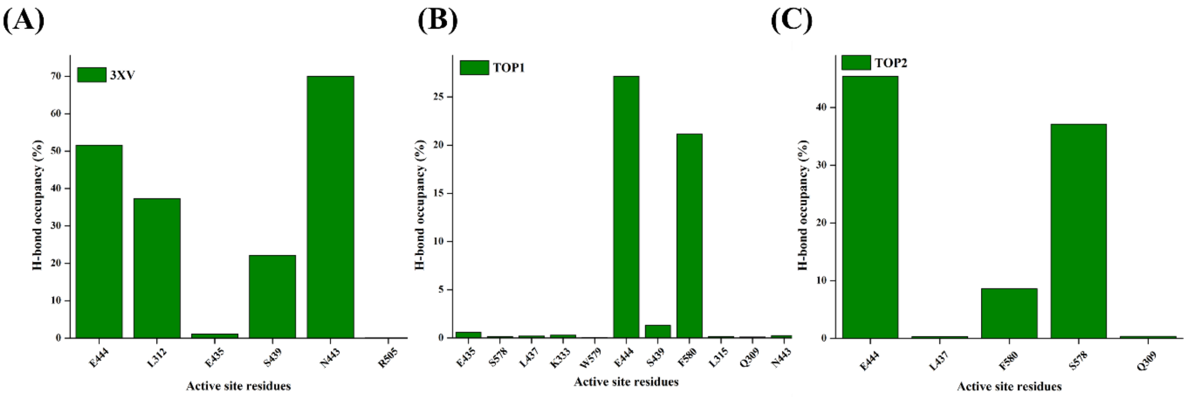

Figure S6. (A) Bar diagram showing the H-bond occupancy of the 3XV-PRMT5 complex. **(B)** Bar diagram showing the H-bond occupancy of the TOP1-PRMT5 complex. **(C)** Bar diagram showing the H-bond occupancy of the TOP2-PRMT5 complex.

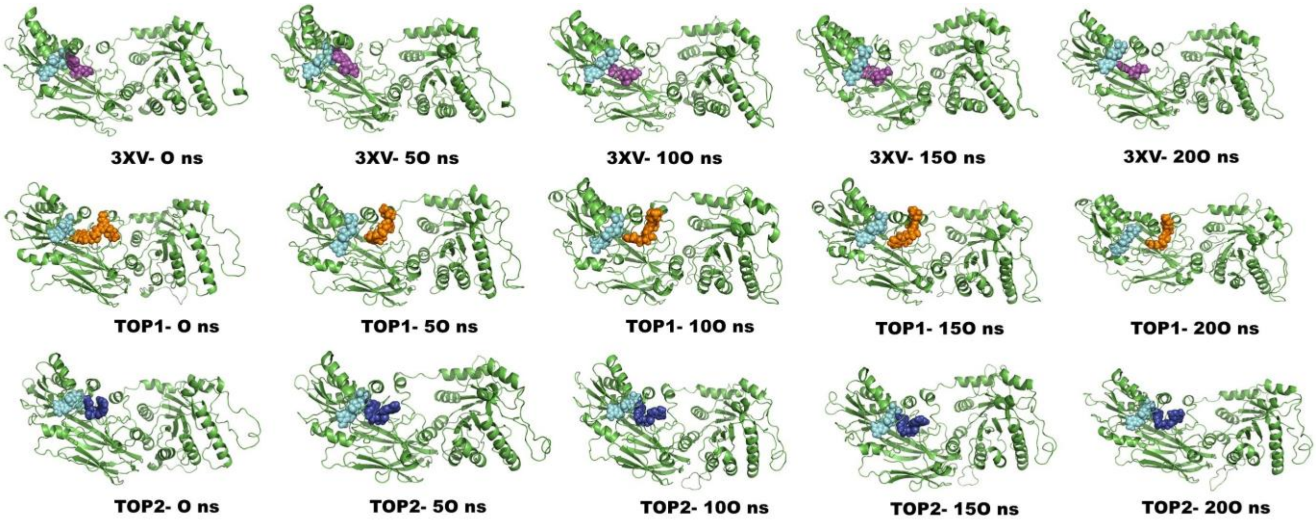

Figure S7. Snapshots of each ligand-bound PRMT5 complex depicting the position of ligands and SAM in the protein at different time intervals observed during the simulation period.
